# Survival of forensic trace evidence on improvised explosive devices: perspectives on individualisation

**DOI:** 10.1038/s41598-020-69385-1

**Published:** 2020-07-30

**Authors:** Natasja Vanderheyden, Elke Verhoeven, Steve Vermeulen, Bram Bekaert

**Affiliations:** 1 Department of Imaging and Pathology, KU Leuven-University of Leuven, Leuven, Belgium; 2 Technical and Scientific Police, Federal Judicial Police, Leuven, Belgium; 3 Department of Forensic Medicine, Laboratory of Forensic Genetics and Molecular Archaeology, University Hospitals Leuven, KU Leuven-University of Leuven, Leuven, Belgium

**Keywords:** Genetic markers, DNA

## Abstract

Improvised Explosive Devices (IEDs) are weapons of modern times, used by terrorist groups and thereby causing substantial damage to communities. There is a widespread misconception that destructive conditions like heat, water or pressure destroy all forensic evidence. Moreover, the plausibility to detect identifiable fingermarks and DNA on components of IEDs is insufficiently known. Therefore, this study investigated the effects of neutralisation and explosion on latent fingerprints and touch DNA. In a majority of the cases, comparative fingermark- and DNA testing resulted in individualisation. In some cases, despite extremely low amounts of contact DNA detected after deployment of render-safe tools or detonation, full STR profiles could be constituted, even after applying fingerprint development techniques. This research shows that latent fingerprints and touch DNA on improvised explosives can be successfully detected after destructive conditions and possibly be linked to the perpetrators of such crimes. This individualising power offers perspectives to enhance forensic investigations of terrorism-related crimes.

## Introduction

The Global Terrorism Index, a measure of the impact of terrorism, describes a decrease in terrorist activity during the last 4 years^1^. Despite the fall in deaths for the third consecutive year, the number of terrorist incidents in 2017 increased to 282 in Europe, up from 253 in the prior year. The impact of terrorism remains widespread, therefore increased counter-terrorism measures must be maintained or even enhanced.

Improvised explosive devices (IEDs) are easy to assemble, homemade explosives, made by unauthorised persons and/or placed in an improvised way^2^. They are composed of at least five key components: a switch, a power source, an initiator, a container and an explosive charge^3^. High explosives like trinitrotoluene (TNT) and C-4 release an enormous amount of energy once detonated, which produces three major effects: a shock wave that distances circularly from the point of origin, release of heat up to 5227 ${^\circ }$C and fragmentation of the explosive device^4^. The national explosive ordnance disposal team evaluates every case individually as a threat, thereby estimating risk ratios to choose the most suitable approach. Above all, maintaining safety of every individual is a primary endpoint, especially in the case of IEDs, in which the design and characteristics are mostly unknown. If possible, human exploration is limited using radio-controlled devices (waterjet disruptors) to examine suspicious objects. In case the IED has not yet exploded, a ballistic water jet can be used to disrupt electrical circuits, thereby neutralizing the IED. Another approach is to destroy the explosive charge, either by executing a controlled explosion or burning.

In the past, terrorism incidents were approached by disciplines as dactyloscopy and explosive residues, often with the assumption that complementary DNA analysis would not be of added value because of the destructive effects of neutralisation and explosion on biological traces^5^. When evidence is subjected to forensic investigation, potential interference occurs between different investigation types that have a destructive effect on forensic evidence. A lack of extensive studies on the plausibility of successful, sequential forensic investigations on IEDs emphasizes the need for extensive research and the development of effective standard operating procedures.

Dactyloscopy, the study of ridge details in fingermark impressions, can be performed to identify the handlers of an object. It is essential for establishing identity in criminal investigations to detect and develop initially invisible marks, also called latent fingerprints. Different fingerprint (FP) enhancement techniques are known whereof the choice depends on the initial composition of the fingermarks, the substrate nature and environmental conditions^6,7^. Previously, exposure to heat and pressure was stated to increase the speed at which the quality of ridge detail deteriorates^8^. Enhancement of latent FPs was seen to be less successful as the duration of exposure to water increased up to 10 days^9^. Cyanoacrylate (CA) fuming is a commonly used development technique based on the interaction of CA with eccrine components of FP residues and the formation of CA polymers. A white deposition is formed on the FP residues and can be subsequently enhanced by the application of a fluorescent dye like Basic Yellow 40 (BY40). The application has been proven successful to develop prints on non-porous surfaces exposed to temperatures up to 500 ${^\circ }$C^8^. Wet surfaces can be treated as well, taken into account that these have to be dried first^10,11^. A previous study applied this to bomb cars subjected to deployment of render-safe tools with promising results^12^. In addition, CA does not interfere with subsequent DNA analysis on post-blast IED fragments^5^.

Another approach is to enhance latent prints by applying wet powder suspensions, which are primarily used on adhesive tape of non-porous surfaces^13,14^. The working mechanism is based on an interaction between the fat soluble components in dermal traces and the hydrophobic tails of the reagents. Characteristic examples are Black Wet Powder (BWP) and Small Particle Reagent (SPR). The latter consists mostly of molybdenum disulphide combined with a surfactant^10^.

Similarly to FP identification, the probative value of DNA in the legal system is based on the phenomenon of genetic variation between individuals of a population^15^. Increased sensitivity of DNA analysis kits for Short Tandem Repeat (STR) sequences allows for reliable and full DNA profiling of samples with just a few cells. Over the years, an alarming trend has been observed whereby an increased capacity of explosives is used by terrorists, rendering the need of DNA analysis as a principal means of identification in disaster perpetrator identification^16^. The success of DNA profiling is negatively affected by low amounts of DNA, degradation and/or PCR inhibitors^17^. Due to technical variation encountered at low amounts of DNA, use of low template DNA (ltDNA) requires careful interpretation of obtained results. These type of analyses are often associated with artefacts: allele drop-in or drop-out, heterozygote peak imbalance and elevated stutter frequencies^18^, resulting in fluctuating results throughout different analyses. Thermal degradation caused by exposure to high temperatures, resulting in cleavage of covalent bonds within DNA strands, has been observed earlier but did not preclude DNA profiling post-blast depending on the type and amount of explosive^19^.

In this paper, a range of IEDs were assembled based on a realistic configuration and subjected to neutralisation or detonation, followed by a successive FP- and DNA analysis on the fragments. Based on this, the probabilities of successfully retrieving latent FPs and DNA after these types of neutralisation and detonation were determined. Ultimately, the perspectives on individualisation of perpetrators after such destructive conditions could alter, leading to an enhanced, adapted and multidisciplinary approach of all actors present on terrorist crime scenes.

## Results

### Exploratory research

The first objective was to identify the most suitable latent FP development technique after exposure to destructive conditions (e.g. water). In this explorative phase, IEDs were exposed to water followed by the performance of three techniques (BWP, SPR or CA-BY40). Visual inspection of the manipulated metal cans resulted in the data shown in Supplementary Table S1. FPs were included if they showed clear ridge details, based on the guidelines of the International Fingerprint Research Group^20^. An analysis of variance demonstrated a significant difference in performance between the three techniques ($p < 0.03$). Further pairwise *t* testing demonstrated a significant difference between CA-BY40 and BWP ($p = 0.01$), in which CA-BY40 displayed significantly better results. Sebaceous FPs could be more easily recovered than natural FPs. The % FP recovery was used as a metric, describing the ratio of the number of detected FPs over the total amount of deposited fingermarks. Examples of visually improved images of developed latent FPs can be found in Supplementary Figure S1.

Based on the findings above, in combination with published literature, CA-BY40 was determined to be the major technique of interest to be used in the experimental research in which latent FPs were exposed to destructive conditions (neutralisation or detonation).

### Positive and negative control

A positive control was included in the experimental design, consisting of an assembled and manipulated IED without successive execution of neutralisation or detonation. The negative control consisted of an assembled, manipulated IED followed by decontamination with RNase Away, also lacking the execution of neutralisation or detonation. Both controls were subjected to comparative FP and DNA analysis.

#### Comparative fingerprint analysis on control IEDs

On the positive control, all initially deposited FPs could be detected after development with CA-BY40 or WWP, corresponding to a FP recovery of 100%. The majority of these FPs was of high quality (score 3), useful for identification and linked to the corresponding reference FPs. The data is shown in Supplementary Table S2. This represents the effectiveness of the FP development techniques. No FPs were detected on the negative control, nor after applying the described FP development techniques, thereby confirming the effectiveness of the decontamination procedure (Supplementary Method S1).

#### Comparative DNA analysis on control IEDs

DNA quantification was performed on all components of the control IEDs. The results can be found in in Supplementary Table S3 and were interpreted according to Supplementary Table S4. The positive control demonstrated characteristic amounts of DNA in a range of 4–70 pg/$\upmu$l on eight out of nine components. One component, the tape, lacked traceable DNA quantities. STR profiling was performed on all eight remaining samples, followed by a comparative DNA analysis. Nothing but full DNA profiles without drop-out phenomena were obtained, representing the ability to recover full STR profiles from touch DNA. The DNA results supported the hypothesis that the involved participant contributed to the biological trace with an extremely strong degree of probability. No traceable amounts of DNA were present on the components of the negative control, reconfirming the effectiveness of the decontamination procedure (Supplementary Table S3).

### Survival of trace evidence after deployment of a waterjet disruptor on IEDs

**Figure 1:**
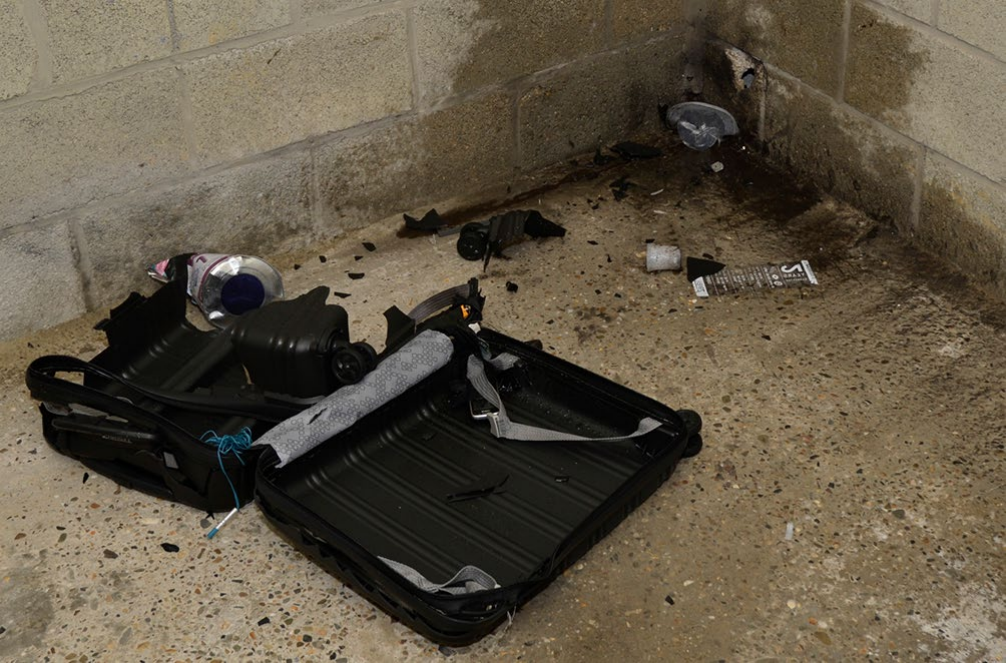
Post-neutralisation experimental setup. To disarm the IEDs, an RE70 M3 Plus Waterjet Disruptor was used, firing a ballistic water jet onto every IED. Variable destructive effects were observed throughout the five testings.

In the first experiment of the experimental research, five IEDs were consecutively disarmed with a RE70 M3 Plus Waterjet Disruptor. The destructive effect was threefold: (partial) destruction of the IED, relocation of the components in the vicinity and humidification of the environment (Fig. 1). All components could be retrieved, except for the push button in the first testing. In the second testing, the suitcase remained nearly intact on the outside due to a less powerful pressure build-up in the disruptor causing the same volume of water to be fired on the suitcase at lower pressure. This resulted in the IED being less exposed to the water.

#### Comparative fingerprint analysis after neutralisation

**Figure 2:**
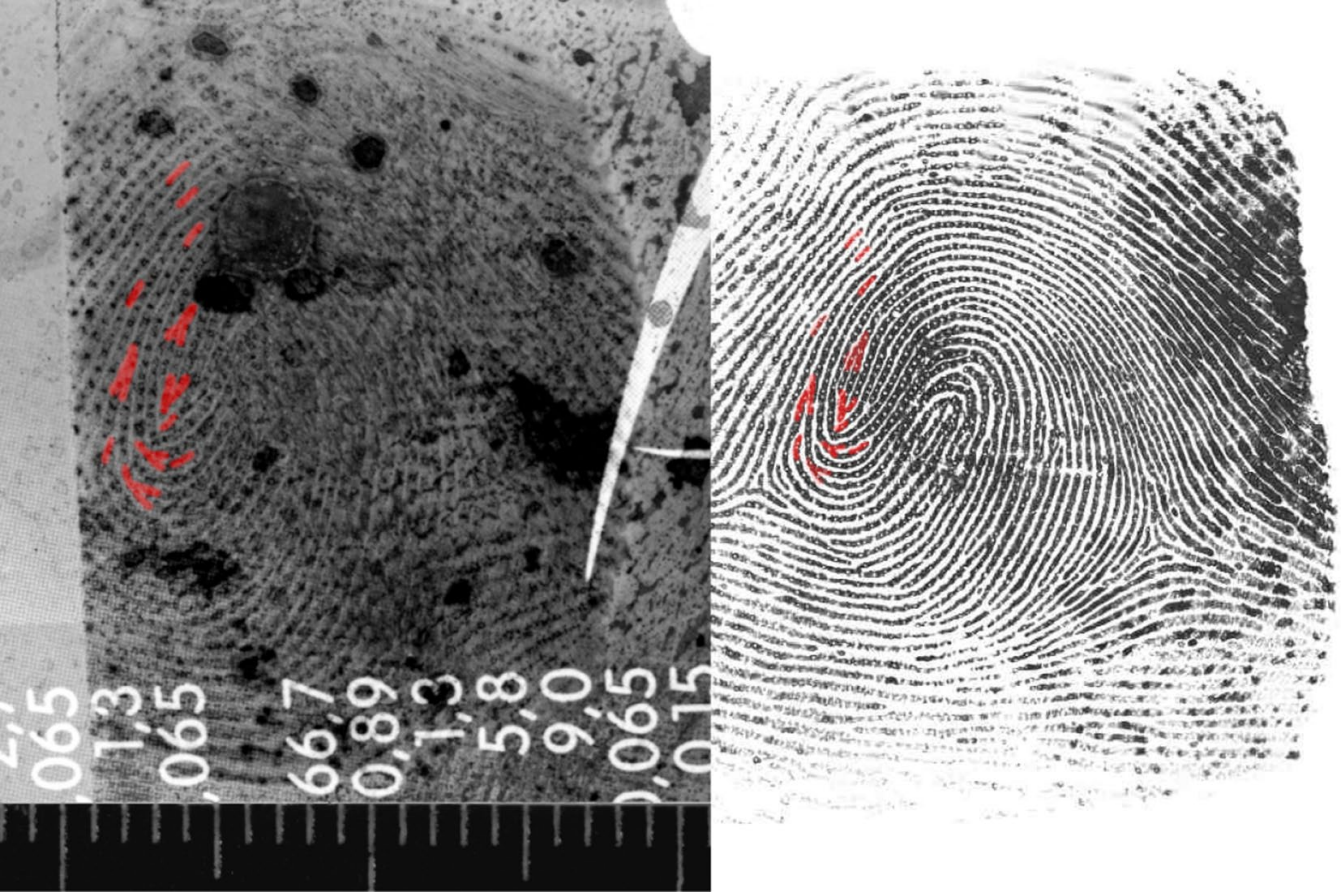
Example of a successful comparative FP analysis after neutralisation. The latent FP (left) was developed with CA-BY40 and photographed, after which 12 corresponding minutiae were marked in red on both the latent- and the reference FP (right).

**Figure 3:**
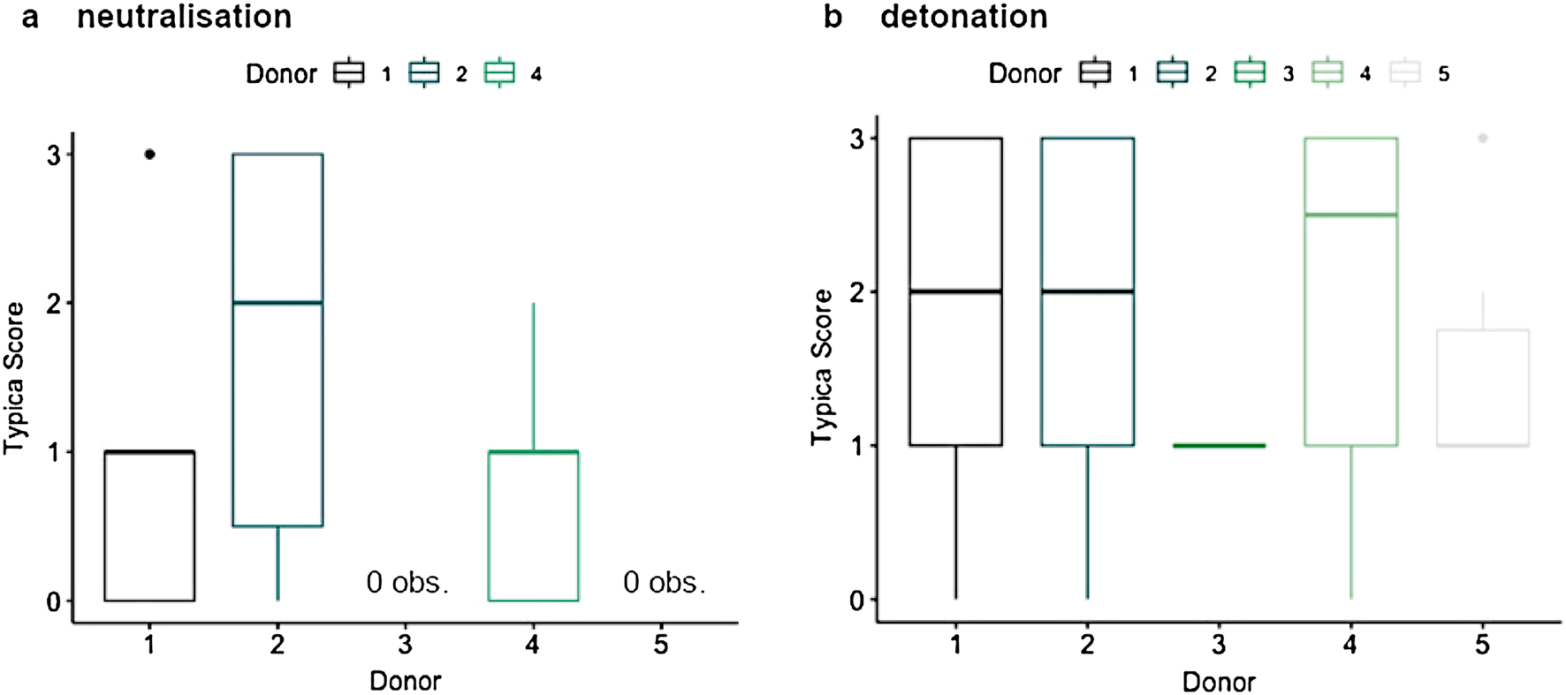
Fingerprint typica scoring after (**a**) neutralisation or (**b**) detonation of IEDs. Scores of latent FPs are based on the number of corresponding typica (minutiae) in the latent FP versus the reference FP for the different individuals. Donors 3 and 5 showed no or low observations in either neutralisation and detonation respectively. No significant difference was seen between the different participants and their typica scores for either neutralisation or detonation.

After neutralisation, latent FPs could be successfully developed with CA-BY40. The results can be found in Supplementary Table S5. Similarly to the exploratory research, a FP recovery was used. 31 of the 115 deposited FPs were detected after deployment of the waterjet disruptor, corresponding to a total FP recovery of 27%. No FPs could be developed and/or detected from donors 3 and 5. Nevertheless, various latent FPs were recovered from donors 1, 2 and 4. The results are shown in Supplementary Table S6. The FP quality was quantitatively scored based on the number of minutiae. 10 FPs turned out to be of poor quality (score 0), not suitable for comparison with reference FPs. 10 prints were of substandard quality (score 1), 3 prints were useful for comparison with reference FPs (score 2) and 8 prints were of high quality, thereby eligible for identification (score 3). An example of a successful match between a latent FP and the reference print after neutralisation can be found in Fig. 2. Figure 3a shows the distribution of typica (minutiae) scores relative to the participants of the neutralisation experiment. Donors 3 and 5 left FPs of apparently inferior quality. Apart from that, incidentally deposited latent FPs on adhesive sides of tape were developed with White Wet Powder (WWP). This resulted in the detection of four FPs: twice score 1 and once score 2 and 3. Donor 1 could be re-identified by this supplementary approach.

#### Comparative DNA analysis after neutralisation

After deployment of the waterjet disruptor, samples were taken from clearly visible FPs and commonly touched areas on components. Results of the DNA quantification process can be found in Supplementary Table S3. In general, the procedure was less successful for donors 3 and 5, in this case corresponding to a lack of detectable latent FPs on their IEDs after neutralisation. Low standard deviations indicate uniformly low amounts of DNA present on the components after neutralisation, except for a couple of outliers observed with donors 2 and 4, whereby larger quantities of DNA were retrieved on the suitcases. The total amount of DNA quantified after neutralisation was not significantly different among the participants ($p > 0.05$).

Subsequently, the amount of DNA per component was analyzed and proven to be significantly different depending on the type of component (One-Way ANOVA, $p = 0.02$, see Supplementary Table S7). Significantly higher amounts of DNA were present on the suitcases compared to the detonators, batteries, metal cans and tape (Post hoc Tukey-adjusted test, $p < 0.05$, see Supplementary Table S8). No significant interaction effect was demonstrated among the components and participants, implying that the detected amounts of DNA on the various components were not individual dependent (Two-Way ANOVA, $p > 0.05$). Moreover, the PowerQuant Kit determined the extent of DNA degradation. This is described with a Degradation Index (DI) metric and evaluated based on the ratio of DNA concentrations determined with autosomal- and degradation targets ([Auto]/[D] ratio). A DI higher than 2 suggests light to moderate DNA degradation of the sample, ratio’s higher than 10 indicate strongly degraded DNA. The stated values are merely indicative and were experimentally determined in an internal validation study (data not provided). [Auto]/[D] ratio’s were calculated for all samples with a mean ratio of 11.20 (n = 8) in a range from 4.29 to 37.46, corresponding to respectively the lowest- and highest DI values. No significant correlation was demonstrated between the DI and the integrity of the STR profiles ($p > 0.05$).

In case the samples contained a larger amount of DNA than the experimentally determined cut-off of 3 pg/$\upmu$l, STR profiling following DNA quantification was carried out. In nine of the 44 samples this was the case and STR profiles were generated. Results of the comparative DNA analysis can be found in Supplementary Table S9 and were interpreted according to Supplementary Table S4. Five out of nine results at least strongly support the hypothesis that the involved participant is the donor of the biological trace, rather than a genetically unrelated individual. The four remaining DNA profiles were insufficiently informative to support the hypothesis that the individual contributed to that particular trace. The latter results were derived from incomplete STR profiles due to allele- or locus dropout and were observed three times originating from mobile phones, once from a suitcase.

### Survival of trace evidence after detonation of IEDs

**Figure 4:**
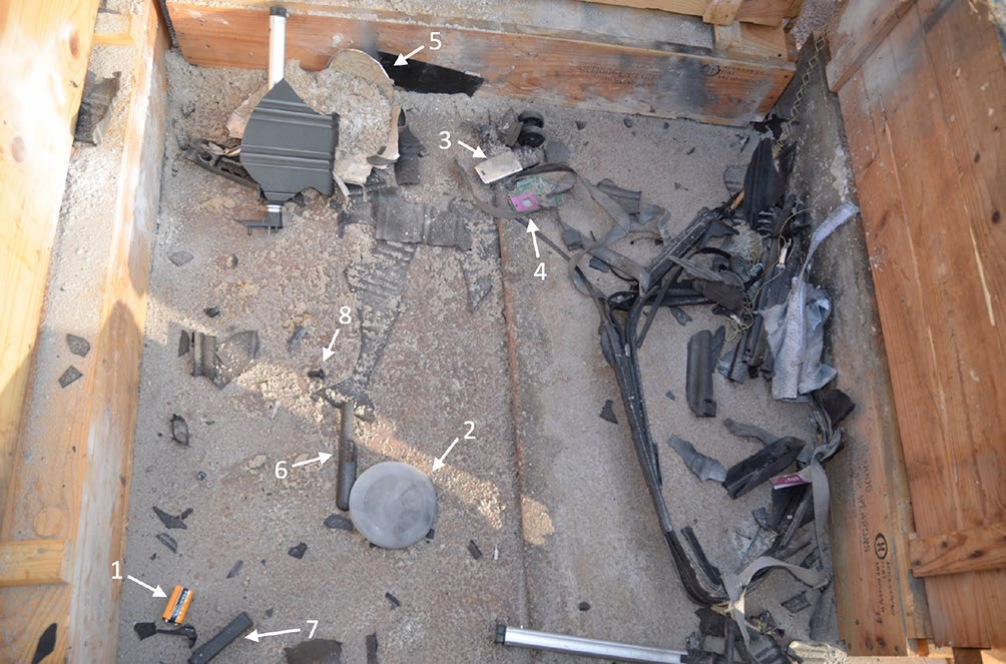
Post-blast experimental setup. Five IEDs were consecutively detonated with 7 g C-4. The arrows mark a few parts of recovered components: (1) battery, (2) lid of metal can, (3) mobile phone, (4) printed circuit board, (5) metal can, (6) handle and (7) combination lock of suitcase, (8) push button.

In the second experiment of the experimental research, five IEDs were consecutively exploded with 7 g C-4. A considerable amount of debris was present, the destructive effect manifested itself to varying degrees. Throughout the successive repetitions there was no consistent scale of damage, different degrees of fragmentation and distortion were observed. Figure 4 shows an example of such a post-blast experimental setting. All components were found and separately collected in labelled paper bags, with the exception of the push buttons from testings 1 and 3 which could not be retrieved.

#### Comparative fingerprint analysis after detonation

Post-blast latent FPs could be successfully developed with CA-BY40. Occasionally, FPs were already visible before application of the development technique due to spontaneous dust- and soot sediments near the explosive charge. After detonation, 52 of the 115 deposited FPs were detected, corresponding to a total FP recovery of 45%. FPs of all five participants could be developed and/or detected after detonation. Thus, the described techniques proved to be convenient for developing latent FPs after exposure to destructive conditions like heat and pressure build-up upon detonation. Results of the comparative fingerprint analysis after detonation can be found in Supplementary Table S10. Quality assessment of the FPs demonstrated 6 prints of poor quality (score 0), not suitable for comparison with reference FPs. 19 prints were of substandard quality (score 1), 7 prints were useful for comparison with reference FPs (score 2) and 20 prints were of high quality, thereby eligible for identification (score 3). Figure 3b shows the distribution of typica (minutiae) scores relative to the participants of the explosion experiment. A clear distribution in the number of detected minutiae is seen. Donors 3 and 5 left FPs of apparently inferior quality.

In addition, incidentally deposited latent FPs on adhesive sides of tape were developed with White Wet Powder (WWP). This resulted in the detection of two FPs, respectively of score 1 and 3. Donor 2 could be re-identified by this approach.

#### Comparative DNA analysis after detonation

Results of the DNA quantification process can be found in Supplementary Table S3. Low standard deviations indicate uniformly low amounts of DNA present on the components after explosion, except for a few outliers observed with donors 2, 4 and 5, whereby larger quantities of DNA were retrieved on the mobile phones. The total amount of DNA quantified after explosion was not significantly different among the individuals ($p > 0.05$). Subsequently, the amount of DNA per component was not significantly different depending on the type of component (One-Way ANOVA, $p > 0.05$, see Supplementary Table S7). Once again, the extent of DNA degradation was described by a Degradation Index (DI). Only two [Auto]/[D] ratio’s could be calculated in this experiment (5.82 and 18.62), potentially resulting from an impaired amplification of the degradation markers.

STR profiling following DNA quantification was carried out according to the same cut-off used in the previous experiment. In eight of the 43 samples it was possible to generate STR profiles. Results and interpretation of the comparative DNA analysis can be found in Supplementary Table S9. Seven out of eight DNA results at least moderately support the hypothesis that the individual is the donor of the biological trace, rather than a genetically unrelated person. The remaining DNA profile was insufficiently informative to support the hypothesis that the participant contributed to that particular trace, resulting from an incomplete STR profile due to allele- or locus dropout. The latter sample was originally derived from a mobile phone.

### Interdisciplinary comparison of the experiments

**Figure 5:**
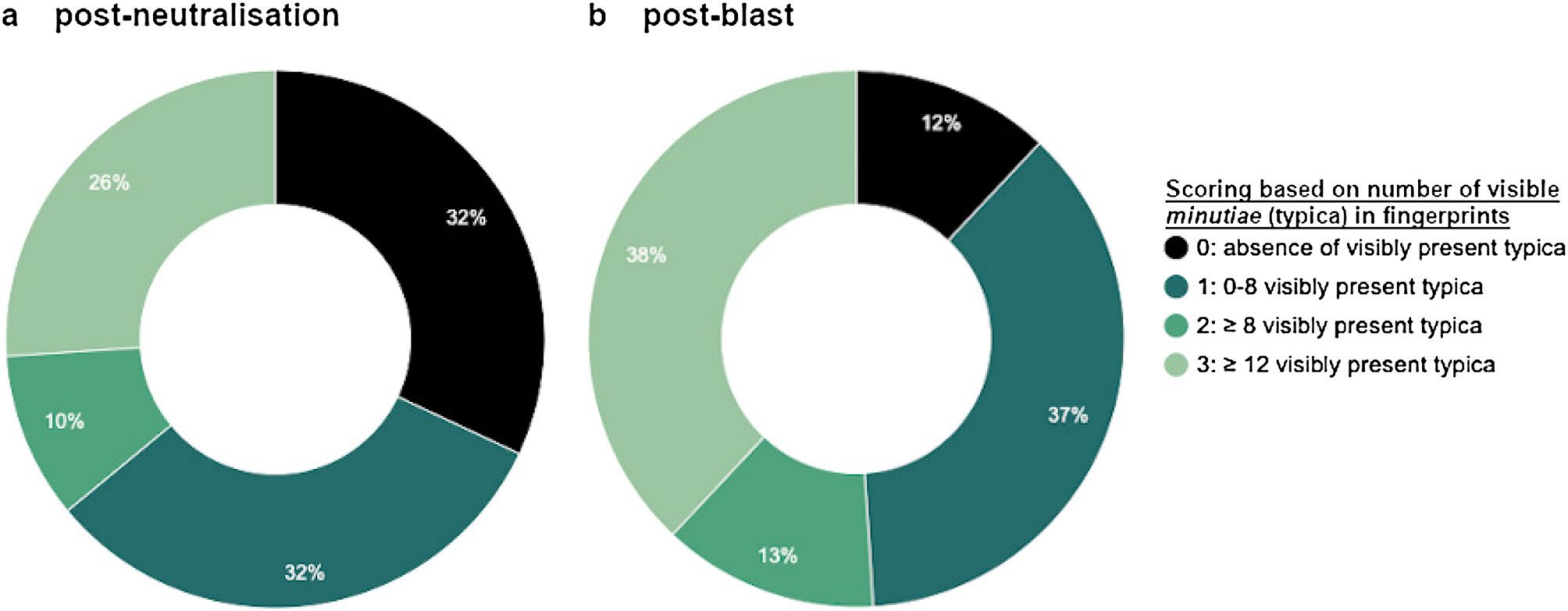
Comparison of typica scores relative to the type of experiment. In both experiments, identifiable fingerprints of score 2 and 3 were detected. However, the percentage of detected fingerprints of high quality was lower after neutralisation (**a**) than after explosion (**b**)

Both after neutralisation and explosion, adequate (latent) FPs were detected for identification, i.e. with a scoring of 2 or higher. The average percentage of FPs detected after execution of the experiments was higher after explosion than after neutralisation. However, the differences between both experiments were not significant ($p >0.05$). Greater fractions of high quality FPs were demonstrated post-blast as compared to post-neutralisation (Fig. 5). The majority of the latent FPs detected after detonation belonged to categories 2 and 3, corresponding to reports “appears to originate from” and “originates from”, respectively, hereby expressing the likelihood that the dermal trace is derived from the corresponding reference FP.

In addition to the promising results concerning the comparative FP analysis, highly distinctive results were obtained in the comparative DNA analysis. In all cases, low template DNA was encountered, i.e. lower than 100 pg. Nevertheless, sufficiently large amounts of DNA were collected from the components to facilitate DNA profiling. A comparative analysis indicated significant differences between the amount of DNA recovered from two types of components after explosion versus neutralisation: metal can and tape ($p < 0.05$). After neutralisation, more DNA was present on the metal can, in contrast to the tape on which more DNA was detected after explosion. A trend towards significance was observed for the suitcase in which more DNA was detected after neutralisation than after explosion ($p = 0.06$). Numeric values can be found in Supplementary Table S3.

70% of the STR profiling attempts resulted in a positive outcome, implying that the obtained DNA results at least moderately support the hypothesis that the involved participant is the donor of the biological trace. These DNA results were derived from a printed circuit board, mobile phone, suitcase, push button or the lid of the metal cans (see Supplementary Table S9). Despite various mixed STR profiles with additional alleles were observed, which could be originating from various sources during assembly of the IEDs until DNA analysis, there was only a limited impact on the results. This was due to a high discriminatory power resulting from multiple analyses and samples, complemented by careful interpretation of the STR profiles.

## Discussion

It appears that improvised explosives (IEDs) will remain the preferred weapon of extremists worldwide throughout the following decades^21^. The variability in technological complexity and manifestations of IEDs create challenges for governments. In the past, IEDs were investigated for the presence of biological traces, but only few research has previously been performed combining the detection of both fingerprints and DNA to identify the involved^5,12^. Moreover, there is no consistency in guidelines describing the most suitable techniques for latent fingerprint development after explosion or neutralisation^5,12,22^. Considering the global social impact of terrorism, it is advisable to optimise forensic investigations and thereby increasing the degree of success.

After deployment of the waterjet disruptor, 60% of the individuals could be identified based on comparative FP and DNA testing, after explosion the degree of identification was even higher, namely 80%. Individuals that were initially identified based on comparative FP analysis, were consecutively identified by comparative DNA analysis, except for one individual in the detonation experiment who could only be identified based on latent FPs (Table 1). Only one individual could not be identified throughout the experiments. This may be a result of extremely degraded DNA and/or a bad shedder status^23^.

**Table 1:** Overview of identifications after neutralisation or explosion.

Donor (reference)	Neutralisation		Explosion	
	FP	DNA	FP	DNA
1	$\checkmark$	$\checkmark$	$\checkmark$	$\checkmark$
2	$\checkmark$	$\checkmark$	$\checkmark$	$\checkmark$
3	0	0	0	0
4	$\checkmark$	$\checkmark$	$\checkmark$	$\checkmark$
5	0	0	$\checkmark$	$\checkmark$

Two experiments were performed in the experimental study, each IED was subjected to neutralisation or explosion. Comparative FP analysis was positively evaluated if the FPs displayed $\ge 8$ (score 2) or $\ge 12$ (score 3) corresponding typica with the reference FP. Comparative DNA analysis was positively evaluated based on likelihood ratios above 1000. Successful identifications are indicated with a check mark ($\checkmark$), unsuccessful comparisons with a symbol (0).

Previously, Esslinger et al. demonstrated a success rate of 20% on components of pipe bombs after deflagration^24^. The success rate of our study was 60% after neutralisation and 80% after detonation. This is high compared to previously conducted research and emphasises the variability of results depending on the type of IED and charge as well as the increased sensitivity of STR-profiling techniques over the past decade. In case the constituted STR-profiles are insufficiently informative, subsequent mitochondrial DNA analysis can be performed^17^.

Consistent intra- and interindividual differences in amount of DNA and FPs were demonstrated, despite a small sample size. Additional parameters such as shedder status and time frame since FP deposition have previously been suggested to have a significant impact on DNA and FP recovery^23^ and offer perspectives for future research. Alternative methods for the collection of biological traces on IED surfaces need to be evaluated, especially on tape and wiring. Previously, the use of an acetone-water DNA recovery protocol was suggested for adhesive sides of electrical tape whereby DNA quantification results would increase up to 70%^25^. It is recommended to further investigate different swabbing tools, wetting agents and substrates^26^.

Notwithstanding that extensive measures were taken in order to mimic real-life scenario’s as much as possible, certain variables were out of our control due to security and costs. A first limitation of the current study is the use of a low amount of C-4 (7 g) compared to real-life cases in order of magnitude in which terrorists often use a larger amount of explosive. This difference has an effect on the temperature developed during the blast^19^ and subsequently on the possibility of recovering FPs and DNA from the IED components^18,27,28^, as well as on the fragmentation of the IED itself^29^. A higher amount of explosive implies a more challenging recovery of FPs and potential DNA carrier components. We therefore acknowledge that the results produced in the current study could be overestimating the chances of producing FP and DNA-profiles compared to real-life scenario’s. However, it must be noted that due to the complex nature of explosions, it is not yet possible to easily predict the magnitude of the blast effects^29^. A second limitation is the limited number of cans detonated and neutralised in the study. To keep variables as constant as possible a strict protocol for assembling the IEDs was followed by the participants. This trade-off assures that the results produced from the experiments are robust though limited in sample size. Finally, it should be noted that the analysis was conducted using miniSTRs. Therefore, the success rate of DNA analysis may not be broadly applicable to standard DNA testing that does not use these types of markers.

Prioritizing cases in which biological traces have been exposed to destructive conditions like pressure, heat and/or water, could improve the preservation and detection of biological material. Sequential practice of multiple forensic disciplines and methods requires prior consideration concerning the trade-off between the expected chance of success and the destructive effect on trace evidence. It is recommended to develop, document and photograph latent FPs prior to DNA sampling and analysis, considering the destructive effect of DNA sampling on FP residues.

This paper demonstrates successful detection of latent FPs and contact DNA on IEDs after deployment of waterjet disruptors or detonation. CA-BY40 was applied to develop latent FPs on components of IEDs, followed by DNA analysis based on STRs. This sequence turned out to be successful on IED components subjected to destructive conditions. Despite extremely low amounts of touch DNA observed after neutralisation or explosion, sometimes full STR profiles could be constituted, even after the application of FP development techniques. An exception to this concerned DNA profiling of developed latent FPs on tape, in which the sequential analysis yielded no successful results. Such substrates require further research on the performance of sampling methods for DNA extraction. Reduced detection of biological traces is a consequence of destruction of cellular material due to generated heat, pressure and/or water after explosion or neutralisation.

The results strongly support the hypothesis that biological traces can survive after neutralisation or detonation. Biological traces transferred to IED components during assembly can thus be potentially linked to their assembler, provided that necessary preventive measures against contamination and loss of cellular material are taken into account. These findings offer perspective on forensic investigational approaches: all actors on a terrorist crime scene can be aligned with contiguous forensics and costs on additional analyses can be reduced.

## Methods

Reference fingerprints were digitally taken (LiveScan, Steria). Buccal swabs were collected for reference DNA profiling (Prionics kit) and stored at $-20\;{^\circ }$C. Personal protective equipment was used to prevent involuntary transfer of biologic material.

### Research permission and ethics declarations

According to the Belgian law^30^, possession of explosive precursors and/or explosives research are strictly regulated. Therefore, an aid application for non-operational support from the Belgian Ministry of Defence to the police was submitted to the Administrative and Technical Secretariat (SAT). An approval for this project was given on July 31, 2018 by the Minister of Defence and Civil Service. The Belgian explosive ordnance disposal team (DOVO) was responsible for ensuring safety before/during/after the explosions and neutralisations. All precursors and explosives were provided by DOVO. All explosion/neutralisation series were executed by experienced military members according to confidential internal procedures of DOVO.

The study was approved by the Ethics Committee of the University of Leuven. Full informed and written consent from the participants was obtained before the initiation of the study for study participation and for publication of the pictures used in the manuscript. The experimental protocol was approved by the University of Leuven and all methods were performed according to the relevant guidelines and regulations.

### Exploratory research

Two participants were included in the exploratory research. Each donor left three sets of six FPs per visualisation technique on a metal can: three eccrine and three sebaceous prints. The FP composition was allowed to recover prior to the different deposition series. A total of 108 FPs was collected in this study.

Three suitable FP development techniques for non-porous surfaces exposed to water, were tested to identify the most optimal method to visualize FPs objected to destructive conditions^14^. Each technique was repeated three times under the same conditions. In total, nine metal cans were manipulated, treated with the relevant technique and analyzed. To simulate water exposure, an injection syringe was filled with 50 ml of tube water and targeted from a distance of 10 cm onto each can. Each metal can was subject to one relevant FP development technique.

#### Cyanoacrylate fuming + basic yellow 40

Latent FPs were visualized with (CA) fuming according to the standard procedure (Foster + Freeman). Metal cans were placed in a fuming cabin, together with 1.4 g CA. A jar of boiling water (500 ml) was added to improve humidification^31^. The vapour development took place in automatic mode. Overdevelopment of FPs was prevented by regular inspection of the process. Hereafter, objects were placed in a laboratory hood for 24 h to enhance polymerisation of CA monomers.

To improve the development of weak FPs on light surfaces, objects were subsequently treated with a Basic Yellow 40 (BY40) staining (0.2% BY40 in ethanol). The staining was sprayed onto the surface for 1 min and then rinsed with demineralized water.

#### Small particle reagent

A concentrated, black SPR solution from molybdenum disulfide and detergent (tergitol) was prepared and converted into a working solution for spray application according to protocols of Home Office^14^. The working solution was shaken thoroughly and sprayed on the metal cans for 1 min to facilitate interaction with the FP residues. Excess SPR was removed with demineralized water.

#### Black wet powder

A commercially available black powder suspension was thoroughly shaken and applied with a brush on the non-porous surfaces of the metal cans. Excess BWP was removed with demineralized water after an incubation time of 1 min.

### Experimental research

The experimental research consisted of two parts; neutralisation and explosion (detonation). Five participants were included in each experiment, including three males and two females aging 21–38 years. FPs were placed on a porcelain plate and developed with black dactyloscopic powder. Every donor was instructed to deposit a series of 23 FPs on the IEDs to ensure consistency throughout the experiments. A total of 115 FPs was collected per experiment. Every donor assembled two IEDs, respectively for the neutralisation- and explosion experiment. Additionally, a suitcase was manipulated to simulate everyday use. The FP composition was allowed to recover naturally prior to the two deposition series and manipulations. Each set of FPs was stored in a dark, stable laboratory environment for a maximum of 1 week ($18\;{^\circ }$C, 37% RH).

#### Safety precautions

All IED neutralisations/explosions were executed by the Belgian bomb disposal team at abandoned destruction sites. Safety perimeters of 200 m were set prior to the experiments and earbuds were worn throughout the experiments. Please note that the utmost caution is needed and explosion/neutralisation series must be executed by experienced military members.

#### Assembly of improvised explosive devices

**Figure 6:**
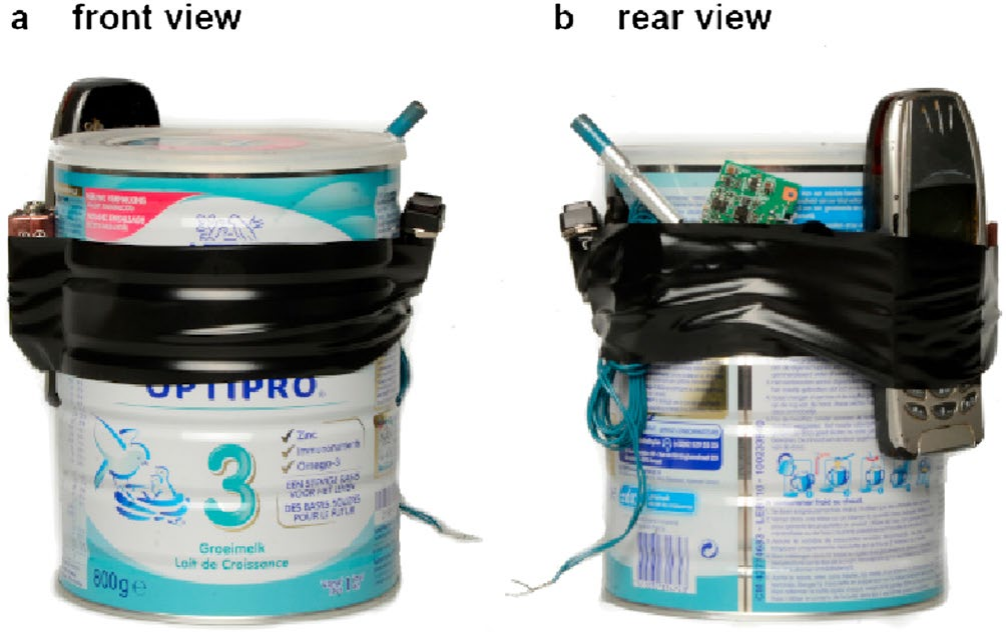
Prototype IED used in the neutralisation and detonation experiments, consisting of the following components: metal can electrical tape, mobile phone, 9 V battery, push button, detonator and a recovered circuit board. In the operational setting, a polypropylene suitcase was used as a container.

Non-porous components and substrates for the assembly of IEDs were intentionally chosen in consultation with DOVO. Every IED consisted of eight components: metal can, polypropylene suitcase, electrical tape, mobile phone, 9 V battery, push button, detonator and a recovered circuit board. A prototype of an intact IED is shown in Fig. 6. The detonation cans were filled with 0.5 l of sand to absorb some energy release during explosion. All substrates were treated with RNase Away according to the decontamination procedure (Supplementary Method S1). The assemblies took place 1 week before the experimental procedure.

Additionally, a positive- and negative control were assembled, consisting of the same parts as the previous ones (IED + suitcase). The positive control was decontaminated and subsequently manipulated, implying the presence of biological traces. The negative control was decontaminated without subsequent manipulation, implying the absence of biological traces. Both controls were not subjected to the destructive conditions of neutralisation or explosion, merely to comparative FP- and DNA analysis.

#### Neutralisation of improvised explosive devices

A ballistic water jet was fired onto the suitcases with a RE70 M3 Plus Waterjet Disruptor (Chemring Technology Solutions). This weapon interrupts electrical circuits without recoil by firing an electrically initiated 0.5 caliber cartridge. This cartridge contains a projected waterjet of 150 ml, fired under high pressure. The weapon was oriented $45{^\circ }$ with the longitudinal surface of the suitcase. After each disarmament, fragments were collected and packaged separately. A test setup of this experiment can be found in Supplementary Figure S2.

#### Detonation of improvised explosive devices

Prior to the experiment, a safety perimeter of 200 m was set. A measured amount of C-4 (7 g) was placed in the metal cans and the suitcases were closed with a zipper. The ignition was electrically initiated from a distance. The presence of wooden crates around each side of the test setup prevented the spread of post-explo fragments. Relocation of the test setup after every explosion prevented cross-contamination. After each detonation, fragments were collected and packaged separately. The test setup of this experiment can be found in Supplementary Figure S3.

### Comparative fingerprint analysis

Latent FPs on IEDs were developed with CA fuming and stained with BY40. To limit degradation of FPs and DNA, all traces were visualized within a 2-week time frame. The electrical tape was removed cautiously and stuck to a transparent foil so FPs could be developed on non-adhesive sides. Latent FPs on adhesive sides were subsequently visualized using White Wet Powder (WWP). The powder suspension was poured into a new dish every time to prevent cross-contamination. The adhesive side of the tape was submerged in the suspension for 3 min and subsequently rinsed with demineralized water.

Developed latent FPs were metrically photographed in situ (100 mm lens, Canon EOS 5D). All original photographs were saved. Metal cans were cut and dented with tin scissors on poorly visible spots. A forensic light source was used (470 nm) and developed FPs were visualized with a yellow filter. Digital improvement of contrast was carried out without creating new pixels (Photoshop CS4).

A comparative FP analysis was performed according to the ACE-V method^32^. Dermal traces were assessed for the presence of minutiae. A digital comparison of visible minutiae between the dermal trace and reference FP was made manually. Traces could be excluded according to three basis patterns (level 1 details), followed by a comparison of minutiae on traces and reference FPs (level 2 details). Evaluation and reporting of the FP analysis was based on a decisive table for FP analysis (Supplementary Table S11). 12 or more corresponding minutiae, without inexplicable differences, individualized a dermal trace. Casual features like scars, warts and skin folds were not taken into account. The same goes for level 3 details, including pores and shapes of papillary lines. In case of uncertainty, analyses were verified by a second expert.

### Comparative DNA analysis

Samples were taken using moistened cotton swabs and immediately stored at $-20\;{^\circ }$C. Semi-automatic DNA isolation of biological traces was executed using the DNA IQ Casework Pro Kit (Promega) for Maxwell 16. Preliminary, a working solution ($90\;\upmu$l incubation buffer, $10\;\upmu$l Proteinase K (20 mg/ml)) was added to each sample, followed by incubation in a thermo block (1 h, $56\;{^\circ }$C). Further steps in the protocol of DNA extraction were according to manufacturer’s instructions, except for a slightly increased amount of elution buffer ($50\;\upmu$l). Extracted samples were stored at $4\;{^\circ }$C. A positive and negative control were included in the DNA isolation process. The positive control contained 0.5 cm of a blood stain on an UltraStain Card. A sterile cotton swab was added as a negative control.

DNA quantification of biological traces was executed using the PowerQuant Kit (Promega) according to manufacturer’s instructions. 96-well plates were prepared using an automated working station JANUS (PerkinElmer) and ran on a 7500 Real-Time PCR System (Applied Biosystems) according to manufacturer’s instructions.

After quantification, all samples containing at least 3 pg/$\upmu$l DNA, were amplified. 22 out of 107 samples met this criterion. Nine variable markers (miniSTRs) were amplified, including sex determination, in an in-house multiplex PCR: D18S51, D22S1045, D10S1248, D21S11, D1S1677, D2S441, FGA, D1S1656 and D12S391. A mastermix was prepared containing $2 \times$ Multiplex PCR Master Mix (Qiagen), primermix Miniplex-9 and nuclease-free water in a total volume of 12.5 $\upmu$l. After addition to the samples, DNA was amplified on a GeneAmp PCR System 9700 (PerkinElmer). Samples containing a DNA concentration lower or equal to 12 pg/$\upmu$l underwent 34 cycles, others 30 cycles. Every cycle consisted of the following steps: denaturation for $15{^\prime }$ at $95\;{^\circ }$C, $30{^\prime }$ at $94\;{^\circ }$C, $1{^\prime }$ at $57\;{^\circ }$C, $1{^\prime }$ at $72\;{^\circ }$C and extension for $45{^\prime }$ at $60\;{^\circ }$C. A positive control DNA sample and negative control (nuclease-free water) underwent the whole amplification process as well. All PCR products were purified with the BigDye XTerminator Purification Kit (Applied Biosystems) to remove unused PCR products and salts. Afterwards, 11 $\upmu$l allelic ladder and 0.5 $\upmu$l internal standard (GeneScan 500 LIZ, Applied Biosystems) was added. Nuclear DNA was separated using capillary electrophoresis on an ABI PRISM 3130XL Genetic Analyzer (Applied Biosystems).

Results of DNA profiling were interpreted in LRmix Studio 1.0. The analytical limit for allele peak height detection was set at 50 RFU. The interpretation is based on a model that measures the evidential value of autosomal forensic DNA profiles and takes into account uncertainties in these profiles by allelic drop-out- and drop-in phenomena. The minimal number of contributors to each DNA profile was estimated based on allele counting. Calculations were based on allele frequencies of the Belgian population and are presented as a Likelihood Ratio (LR, Supplementary Table S4).

### Statistical analysis

Statistical analyses (*t*-test and One-way ANOVA) were performed in R Studio, $\alpha = 0.05$ was used as level of significance.

## Supplementary information

Supplementary Information.
