# Supplementary material for: Survival of forensic trace evidence on improvised explosive devices: perspectives on individualisation
Source: Sci Rep. 2020 Jul 30;10:12813. doi: 10.1038/s41598-020-69385-1 (PMC7392899; doi:10.1038/s41598-020-69385-1)
Supplement: Supplementary file 1 — Supplementary Information. [file 41598_2020_69385_MOESM1_ESM.pdf]

Survival of forensic trace evidence on improvised explosive devices: perspectives on individualisation

Authors:
Natasja Vanderheyden[1], Elke Verhoeven[2], Steve vermeulen[2], and Bram Bekaert[1,3]
[1]KU Leuven - University of Leuven; Department of Imaging and Pathology; Leuven, Belgium
[2]Technical and Scientific Police; Federal Judicial Police; Leuven, Belgium
[3]KU Leuven - University of Leuven; University Hospitals Leuven; Department of Forensic Medicine; Laboratory of Forensic Genetics and Molecular Archaeology, Leuven, Belgium

Supplementary methods

Supplementary method S1. Decontamination procedure with RNase Away.

- Spray RNase Away on the surface, incubate for 5 minutes
- Wipe the surface with paper tissues and rinse with water
- Wipe the surface with paper tissues and rinse with 70 % ethanol
- Wipe the surface with paper tissues

Supplementary figures

Supplementary figure S1. Examples of developed latent fingerprints after exposure to water. Latent fingerprints were developed with (A) Black Wet Powder (BWP), (B) Small Particle Reagent (SPR) or (C) Cyanoacrylate - Basic Yellow 40 (CA-BY40).

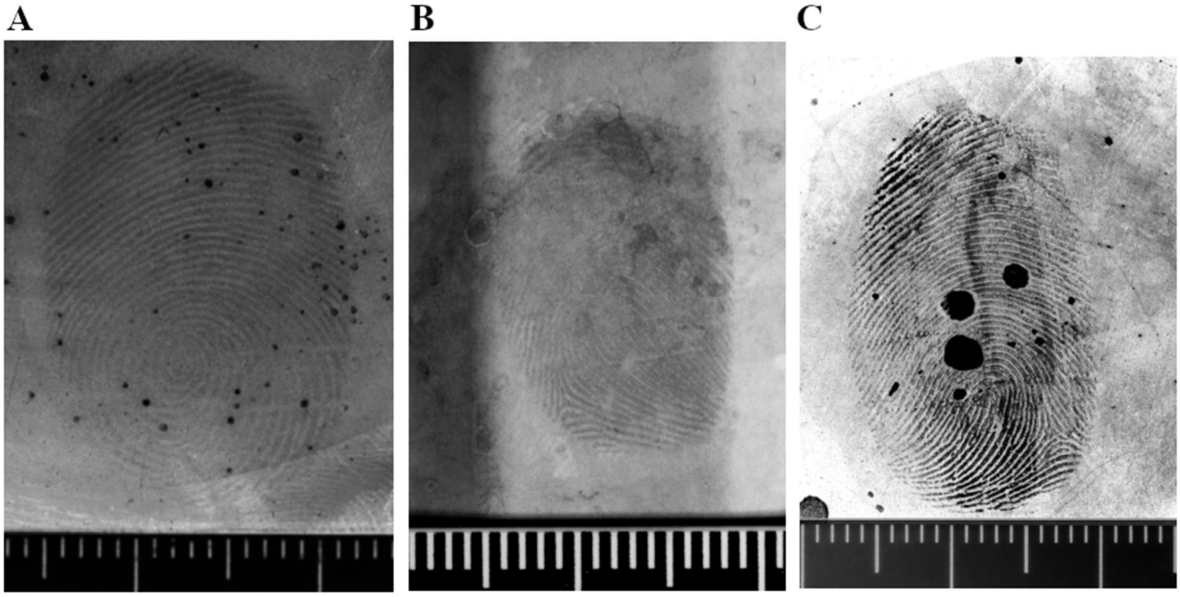

Supplementary figure S2. Test setup experiment 1 – neutralisation of IEDs.
The RE70 M3 Plus Waterjet Disruptor was fired onto a suitcase containing an IED five times under an angle of 45° with the longitudinal surface of the suitcase.

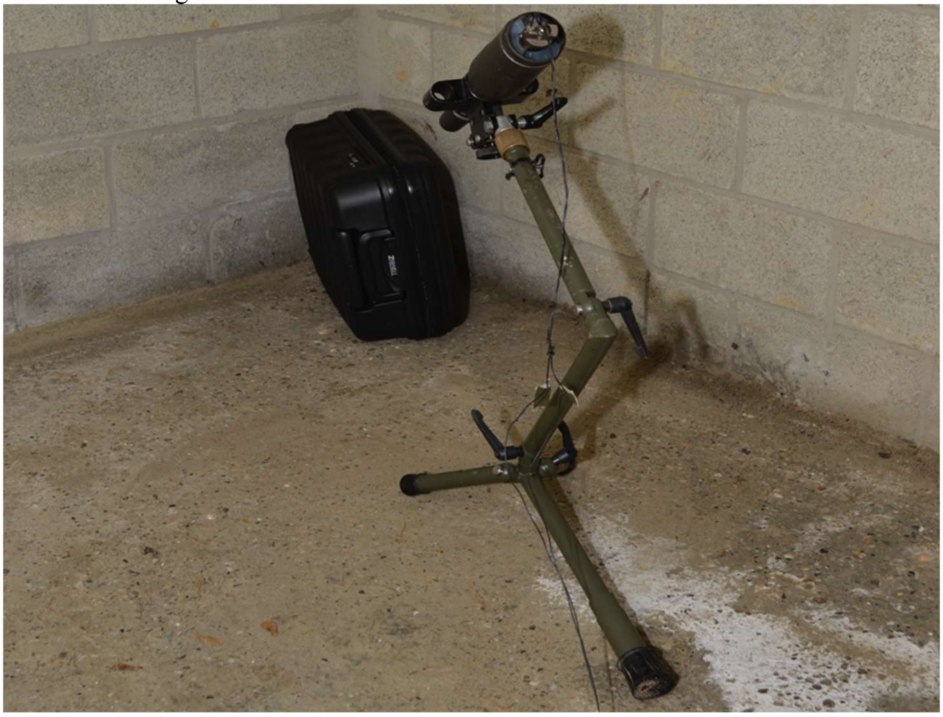

Supplementary figure S3. Test setup experiment 2 – detonation of IEDs.
A prototype of an electrically initiated IED with a charge of 7 g C-4 (9.2 g TNT equivalence).

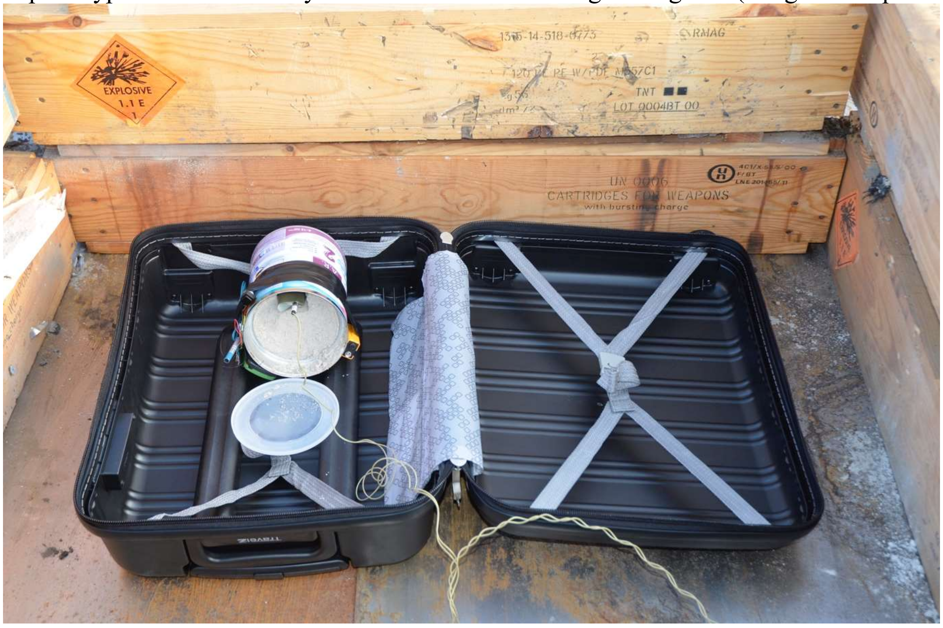

Supplementary tables

Supplementary table S1. Comparison of fingerprint development techniques after exposure to water.

The numeric values state the number of detected fingerprints (FPs) treated with the relevant technique in the exploratory research: Black Wet Powder (BWP), Small Particle Reagent (SPR) or Cyanoacrylate-Basic Yellow 40 (CA-BY40). In total, 108 fingermarks were deposited throughout the three techniques and repeat sequences. The ratio of the number of detected FPs over the total amount of deposited fingermarks is shown as a % FP recovery per donor. S: sebaceous fingerprints; E: eccrine fingerprints.

	BWP		SPR		CA-BY40	
	S	E	S	E	S	E
Repeat sequence 1	2	1	0	0	5	1
Repeat sequence 2	0	0	0	1	4	2
Repeat sequence 3	0	0	4	2	3	3
FP recovery	11 %	6 %	22 %	17 %	67 %	33 %

Supplementary table S2. Results fingerprint analysis positive control.

The ratio of the number of detected FPs over the total amount of deposited fingermarks is shown as a % FP recovery per donor.

Donor	# minutiae	Scoring	Reporting	FP recovery
4	15	3	Originates from...	
4	15	3	Originates from...	
4	13	3	Originates from...	
4	13	3	Originates from...	
4	13	3	Originates from...	
4	14	3	Originates from...	
4	7	1	Unsuccessful query	
4	15	3	Originates from...	
4	8	2	Appears to originate from...	
4	13	3	Originates from...	
4	8	2	Appears to originate from...	
4	19	3	Originates from...	
4	7	1	Unsuccessful query	
4	13	3	Originates from...	
4	12	3	Originates from...	
4	3	1	Unsuccessful query	
4	16	3	Originates from...	
4	6	1	Unsuccessful query	
4	12	3	Originates from...	
4	21	3	Originates from...	
4	12	3	Originates from...	
4	23	3	Originates from...	
4	22	3	Originates from...	100 %

Supplementary table S3. Results DNA quantification.

Testings included in the following table: neutralisation, explosion, positive- and negative control. The ratio of DNA concentrations determined with autosomal- and degradation targets (Auto]/[D] ratio) can be used to evaluate the extent of degradation. PSB: Printed Circuit Board, Lid: lid of metal can, N: neutralisation, E: explosion, C+: Positive Control, C-: Negative Control.

Component	Individual	Experiment	Autosomal DNA	Y-chr (ng/µl)	Autosomal/ Y	[Auto]/[D]
Detonator	1	N	0,0000	0,0000	-	-
Battery	1	N	0,0000	0,0000	-	-
PSB	1	N	0,0043	0,0030	1,4453	6,0182
Mobile phone	1	N	0,0085	0,0037	2,3221	10,6301
Lid	1	N	0,0018	0,0006	2,9260	2,5226
Metal can	1	N	0,0005	0,0002	2,0756	2,4097
Tape	1	N	0,0000	0,0000	-	-
Suitcase	1	N	0,0035	0,0000	-	4,2902
Detonator	2	N	0,0005	0,0001	3,9666	-
Battery	2	N	0,0007	0,0001	4,4239	2,0679
PSB	2	N	0,0043	0,0023	1,8626	9,0796
Mobile phone	2	N	0,0108	0,0063	1,7236	10,6572
Push button	2	N	0,0017	0,0015	1,1319	6,0247
Lid	2	N	0,0007	0,0005	1,5401	-
Metal can	2	N	0,0008	0,0000	-	5,5906
Tape	2	N	0,0000	0,0000	-	-
Suitcase	2	N	0,0502	0,0354	1,4197	4,3850
Detonator	3	N	0,0001	0,0000	-	-
Battery	3	N	0,0001	0,0000	-	-
PSB	3	N	0,0016	0,0009	1,8558	-
Mobile phone	3	N	0,0142	0,0062	2,2974	37,4643
Push button	3	N	0,0000	0,0000	-	-
Lid	3	N	0,0028	0,0011	2,4275	2,5874
Metal can	3	N	0,0001	0,0000	-	-
Tape	3	N	0,0000	0,0000	-	-
Suitcase	3	N	0,0009	0,0004	2,1548	6,0639
Detonator	4	N	0,0021	0,0000	-	2,7260
Battery	4	N	0,0001	0,0000	-	-
PSB	4	N	0,0084	0,0003	33,4404	7,1132
Mobile phone	4	N	0,0026	0,0000	-	11,6985
Push button	4	N	0,0011	0,0000	-	5,0496
Lid	4	N	0,0023	0,0000	-	2,8546
Metal can	4	N	0,0013	0,0000	-	-
Tape	4	N	0,0000	0,0000	-	-
Suitcase	4	N	0,0587	0,0249	2,3619	-

Detonator	5	N	0,0019	0,0000	-	9,5969
Battery	5	N	0,0001	0,0000	-	-
PSB	5	N	0,0015	0,0000	-	6,8062
Mobile phone	5	N	0,0023	0,0004	5,9344	-
Push button	5	N	0,0019	0,0000	-	9,4233
Lid	5	N	0,0003	0,0000	-	-
Metal can	5	N	0,0007	0,0000	-	-
Tape	5	N	0,0000	0,0986	0,0000	0,0000
Suitcase	5	N	0,0012	0,0001	10,3674	-
Detonator	1	E	0,0000	0,0000	-	-
Battery	1	E	0,0008	0,0005	1,7254	-
PSB	1	E	0,0003	0,0001	2,6614	-
Mobile phone	1	E	0,0015	0,0008	1,8977	9,2408
Lid	1	E	0,0001	0,0000	-	-
Metal can	1	E	0,0000	0,0002	0,0000	0,0000
Tape	1	E	0,0000	0,0000	-	-
Suitcase	1	E	0,0018	0,0009	2,1086	9,7927
Detonator	2	E	0,0007	0,0005	1,2698	-
Battery	2	E	0,0024	0,0017	1,4041	7,8534
PSB	2	E	0,0034	0,0021	1,6301	-
Mobile phone	2	E	0,0011	0,0004	2,9464	-
Push button	2	E	0,0033	0,0024	1,3388	5,8237
Lid	2	E	0,0052	0,0051	1,0353	-
Metal can	2	E	0,0000	0,0073	0,0000	0,0000
Tape	2	E	0,0000	0,0000	-	-
Suitcase	2	E	0,0096	0,0073	1,3138	-
Detonator	3	E	0,0000	0,0000	-	-
Battery	3	E	0,0000	0,0000	-	-
PSB	3	E	0,0003	0,0002	1,3466	-
Mobile phone	3	E	0,0001	0,0000	-	-
Lid	3	E	0,0000	0,0000	-	-
Metal can	3	E	0,0000	0,0000	-	-
Tape	3	E	0,0002	0,0000	-	-
Suitcase	3	E	0,0000	0,0000	-	-
Detonator	4	E	0,0008	0,0000	-	-
Battery	4	E	0,0000	0,0000	-	-
PSB	4	E	0,0032	0,0000	-	-
Mobile phone	4	E	0,0084	0,0010	8,0543	-
Push button	4	E	0,0007	0,0000	-	-
Lid	4	E	0,0001	0,0000	-	-
Metal can	4	E	0,0004	0,0000	-	-

Tape	4	E	0,0005	0,0000	-	1,0467
Suitcase	4	E	0,0004	0,0000	-	-
Detonator	5	E	0,0002	0,0000	-	-
Battery	5	E	0,0008	0,0000	-	-
PSB	5	E	0,0052	0,0000	-	18,6215
Mobile phone	5	E	0,0152	0,0033	4,6097	-
Push button	5	E	0,0007	0,0000	-	-
Lid	5	E	0,0002	0,0000	-	-
Metal can	5	E	0,0004	0,0001	4,2384	-
Tape	5	E	0,0000	0,0000	-	-
Suitcase	5	E	0,0002	0,0000	-	-
Detonator	-	C+	0,0071	0,0000	-	8,7104
Battery	-	C+	0,0041	0,0001	46,2413	8,2599
PSB	-	C+	0,0417	0,0006	66,6293	9,1952
Mobile phone	-	C+	0,0172	0,0002	73,5432	6,8436
Push button	-	C+	0,0229	0,0004	62,2658	6,8556
Lid	-	C+	0,0748	0,0002	311,0684	2,4028
Metal can	-	C+	0,0043	0,0003	16,2237	3,9248
Tape	-	C+	0,0000	0,0000	-	-
Suitcase	-	C+	0,0112	0,0001	81,1320	5,2249
Detonator	-	C-	0,0000	0,0000	-	-
Battery	-	C-	0,0000	0,0000	-	-
PSB	-	C-	0,0000	0,0000	-	-
Mobile phone	-	C-	0,0001	0,0000	-	-
Push button	-	C-	0,0000	0,0000	-	-
Lid	-	C-	0,0000	0,0000	-	-
Metal can	-	C-	0,0000	0,0000	-	-
Tape	-	C-	0,0000	0,0000	-	-
Suitcase	-	C-	0,0000	0,0000	-	-

Supplementary table S4. Interpretation of DNA profiles based on likelihood ratios.
Based on internal documentation employed by forensic DNA laboratories in Belgium.

Likelihood Ratio (LR)	The obtained DNA results support…	Probability to observe the DNA profile in a Belgian population
Greater than 1 billion	extremely strong, bordering certainty	≤ 1 in 1 billion
1 million - 1 billion	extremely strong	1 in 1 million - 1 in 1 billion
100.000 - 1 million	very strong	1 in 100.000 - 1 in 1 million
10.000 - 100.000	strongly	1 in 10.000 - 1 in 100.000
1.000 - 10.000	moderately	1 in 1.000 - 1 in 10.000
100 - 1.000	weakly	1 in 100 - 1 in 1.000
	…the hypothesis that the involved subject contributed to the biological trace.	
1 - 100	The obtained results are insufficiently informative.	≤ 1 in 100

Supplementary table S5. Results comparative fingerprint analysis after neutralisation.
The number of detected (latent) fingerprints relative to the originally deposited number of fingerprints is described as the % Fingerprint Recovery per individual.

Individual	# *minutiae*	Scoring	Reporting	FP recovery
1	0	0	Invalidate	
1	0	0	Invalidate	
1	3	1	Unsuccessful query	
1	13	3	Originates from...	
1	3	1	Unsuccessful query	**21,7 %**
2	21	3	Originates from...	
2	0	0	Invalidate	
2	20	3	Originates from...	
2	28	3	Originates from...	
2	0	0	Invalidate	
2	9	2	Appears to originate from...	
2	3	1	Unsuccessful query	
2	3	1	Unsuccessful query	
2	12	3	Originates from...	
2	9	2	Appears to originate from...	
2	24	3	Originates from...	
2	13	3	Originates from...	
2	12	3	Originates from...	

2	0	0	Invalidate	
2	0	0	Invalidate	**65,2 %**
4	3	1	Unsuccessful query	
4	0	0	Invalidate	
4	0	0	Invalidate	
4	0	0	Invalidate	
4	2	1	Unsuccessful query	
4	0	0	Invalidate	
4	2	1	Unsuccessful query	
4	7	1	Unsuccessful query	
4	7	1	Unsuccessful query	
4	5	1	Unsuccessful query	
4	10	2	Appears to originate from...	**47,8 %**

Supplementary table S6. Fingerprint recovery after neutralisation or detonation.
Ten IEDs were experimentally neutralized or detonated in a controlled manner. The number of detected (latent) fingerprints relative to the originally deposited number is described as a % Fingerprint Recovery per experiment.

Individual	*% FP recovery after neutralisation*	*% FP recovery after explosion*
1	21,7	69,6
2	65,2	78,3
3	0,0	8,7
4	47,8	43,5
5	0,0	26,1

Supplementary table S7. Overview DNA quantification according to the type of component after neutralisation or detonation.
Extremely low quantities of DNA (low template DNA) were retrieved on all components, except for the tape. A larger amount of DNA was detected on samples originating from suitcases ($p < 0.05$; $n = 5$). n: number of observations, PSB: Printed Circuit Board, Lid: lid of metal can, N: neutralisation, E: explosion.

Component	Experiment	n	Sum	Mean quantity DNA (ng/µl)	Variance
Detonator	N	5	0,005	0,001	1,0E-06
Battery	N	5	0,001	0,000	6,7E-08
PSB	N	5	0,020	0,004	7,9E-06
Mobile phone	N	5	0,038	0,008	2,7E-05
Push button	N	4	0,005	0,001	7,3E-07
Lid	N	5	0,008	0,002	1,1E-06
Metal can	N	5	0,003	0,001	2,0E-07
Tape	N	5	0,000	0,000	0,0E+00
Suitcase	N	5	0,115	0,023	8,4E-04
Detonator	E	5	0,002	0,000	1,4E-07
Battery	E	5	0,004	0,001	9,4E-07

PSB	E	5	0,012	0,002	4,5E-06
Mobile phone	E	5	0,026	0,005	4,2E-05
Push button	E	3	0,005	0,002	2,2E-06
Lid	E	5	0,080	0,016	1,1E-03
Metal can	E	5	0,001	0,000	5,1E-08
Tape	E	5	0,001	0,000	4,5E-08
Suitcase	E	5	0,012	0,002	1,7E-05

Supplementary table S8. Results statistical analysis after neutralisation.
a) One-Way Anova: A significant difference was observed between the amount of DNA relative to the different type of components df: degrees of freedom.
b) Two-Way Anova with interaction effect: No significant difference was observed between component and individual.
c) Post hoc Tukey-adjusted pairwise comparisons: Pairwise comparisons of the components demonstrate a significant difference in amount of DNA between the suitcase and the following components: detonator, battery, metal can and tape. SE: standard error, df: degrees of freedom.

Contrast	Estimation	SE	df	t.ratio	p value
Detonator - Battery	1.36e-04	0.00364	43	0.037	1
Detonator - PSB	-2.61e-03	0.00364	43	-0.718	0.9983
Detonator - Mobile phone	-5.84e-03	0.00364	43	-1.602	0.7983
Detonator - Lid	-7.22e-04	0.00364	43	-0.198	1
Detonator - Metal can	2.08e-04	0.00364	43	0.057	1
Detonator - Tape	5.67e-04	0.00364	43	0.156	1
Detonator - Suitcase	-1.20e-02	0.00364	43	-3.299	0.0459
Detonator - Push button	-	-	-	-	-
Battery - PSB	-2.75e-03	0.00364	43	-0.755	0.9975
Battery - Mobile phone	-5.97e-03	0.00364	43	-1.640	0.7779
Battery - Lid	-8.58e-04	0.00364	43	-0.236	1
Battery - Metal can	7.19e-05	0.00364	43	0.020	1
Battery - Tape	4.31e-04	0.00364	43	0.118	1
Battery - Suitcase	-1.22e-02	0.00364	43	-3.337	0.0418
Battery - Push button	-	-	-	-	-
PSB - Mobile phone	-3.22e-03	0.00364	43	-0.885	0.9927
PSB - Lid	1.89e-03	0.00364	43	0.519	0.9998
PSB - Metal can	2.82e-03	0.00364	43	0.775	0.9970
PSB - Tape	3.18e-03	0.00364	43	0.873	0.9933
PSB - Suitcase	-9.41e-03	0.00364	43	-2.582	0.2242
PSB - Push button	-	-	-	-	-
Mobile phone - Lid	5.12e-03	0.00364	43	1.404	0.8901
Mobile phone - Metal can	6.05e-03	0.00364	43	1.659	0.7667
Mobile phone - Tape	6.40e-03	0.00364	43	1.758	0.7081
Mobile phone - Suitcase	-6.18e-03	0.00364	43	-1.697	0.7450
Mobile phone - Push button	-	-	-	-	-
Lid - Metal can	9.30e-04	0.00364	43	0.255	1
Lid - Tape	1.29e-03	0.00364	43	0.354	1
Lid - Suitcase	-1.13e-02	0.00364	43	-3.101	0.0742
Lid - Push button	-	-	-	-	-

Metal can - Tape	3.59e-04	0.00364	43	0.099	1
Metal can - Suitcase	-1.22e-02	0.00364	43	-3.356	0.0397
Metal can - Push button	-	-	-	-	-
Tape - Suitcase	-1.26e-02	0.00364	43	-3.455	0.0308
Tape - Push button	-	-	-	-	-
Suitcase - Push button	-	-	-	-	-

Supplementary table S9. Overview DNA analysis and interpretation.
Results from the comparative DNA analysis after neutralisation, explosion and derivations from the positive control (C+) are shown in the table below. LR: Likelihood Ratio.

Experiment	Autosomal DNA (ng/µl)	Integrity STR-profiel	Minimal no. contributors	Individual (reference)	LR	Interpretation comparative DNA analysis
N	0.004	100 %	2	1	1,25E+06	Extremely strong
N	0.008	69 %	2	1	2,18E-11	Excluded
N	0.004	44 %	2	1	3,80E+06	Extremely strong
N	0.004	100 %	2	2	4,59E+05	Very strong
N	0.011	94 %	4	2	2,87E+01	Insufficiently informative
N	0.050	100 %	1	2	2,61E+08	Extremely strong
N	0.014	47 %	1	3	9,88E-08	Excluded
N	0.008	100 %	3	4	2,00E+04	Strong
N	0.059	83 %	2	4	2,77E+01	Insufficiently informative
E	0,003	89 %	2	2	2,40E+04	Strong
E	0,003	100 %	2	2	4,56E+06	Extremely strong
E	0.005	100 %	2	2	2,57E+07	Extremely strong
E	0.010	100 %	2	2	2,70E+07	Extremely strong
E	0,003	83 %	2	4	3,63E+05	Very strong
E	0.008	100 %	3	4	6,90E+03	Moderate
E	0.005	100 %	2	5	1,43E+07	Extremely strong
E	0.015	39 %	2	5	1,49E-02	Excluded
C+	0.007	100 %	1	4	4,98E+10	Extremely strong, bordering certainty
C+	0.004	100 %	2	4	4,98E+10	Extremely strong, bordering certainty
C+	0.042	100 %	1	4	1,05E+11	Extremely strong, bordering certainty
C+	0.017	100 %	1	4	1,05E+11	Extremely strong, bordering certainty
C+	0.023	100 %	1	4	1,05E+11	Extremely strong, bordering certainty
C+	0.075	100 %	1	4	1,05E+11	Extremely strong, bordering certainty
C+	0.004	100 %	2	4	1,05E+07	Extremely strong
C+	0.011	100 %	2	4	1,70E+07	Extremely strong

Supplementary table S10. Results comparative fingerprint analysis after detonation.
The number of detected (latent) fingerprints relative to the originally deposited number of fingerprints is described as the % Fingerprint Recovery per individual.

Individual	# *minutiae*	Scoring	Reporting	FP recovery
1	5	1	Unsuccessful query	
1	13	3	Originates from...	
1	8	2	Appears to originate from...	
1	9	2	Appears to originate from...	
1	11	2	Appears to originate from...	
1	16	3	Originates from...	
1	1	1	Unsuccessful query	
1	6	1	Unsuccessful query	
1	17	3	Originates from...	
1	16	3	Originates from...	
1	6	1	Unsuccessful query	
1	4	1	Unsuccessful query	
1	0	0	Invalidate	
1	0	0	Invalidate	
1	14	3	Originates from...	
1	12	3	Originates from...	**69,6 %**
2	12	3	Originates from...	
2	4	1	Unsuccessful query	
2	16	3	Originates from...	
2	6	1	Unsuccessful query	
2	12	3	Originates from...	
2	12	3	Originates from...	
2	0	0	Invalidate	
2	0	0	Invalidate	
2	10	2	Appears to originate from...	
2	35	3	Originates from...	
2	0	0	Invalidate	
2	12	3	Originates from...	
2	2	1	Unsuccessful query	
2	3	1	Unsuccessful query	
2	11	2	Appears to originate from...	
2	4	1	Unsuccessful query	
2	26	3	Originates from...	
2	17	3	Originates from...	**78,3 %**
3	3	1	Unsuccessful query	
3	2	1	Unsuccessful query	**8,7 %**
4	12	3	Originates from...	
4	1	1	Unsuccessful query	
4	6	1	Unsuccessful query	
4	0	0	Invalidate	
4	5	1	Unsuccessful query	
4	8	2	Appears to originate from...	
4	12	3	Originates from...	
4	13	3	Originates from...	
4	17	3	Originates from...	
4	14	3	Originates from…	**43,5 %**
5	12	3	Originates from...	
5	3	1	Unsuccessful query	
5	4	1	Unsuccessful query	
5	8	2	Appears to originate from...	
5	7	1	Unsuccessful query	
5	5	1	Unsuccessful query	**26,1 %**

Supplementary table S11. Quality assessment and reporting of developed latent fingermarks in comparative fingerprint research.

Scoring of fingerprints based on the number of minutiae and visible ridge details, partly based on guidelines of the Biometric Identification Service (Federal Judicial Police Belgium).

Scoring	Quality level based on the number of minutiae (X)	Reporting
0	/	Unfit
1	$0 < X < 8$	Unsuccessful query
2	$8 \leq X \leq 11$	"Appears to originate from…"
3	≥ 12	"Originates from…"
